# Negative pressure wound therapy: does it suck?

**DOI:** 10.1093/bjs/znaf093

**Published:** 2025-05-06

**Authors:** Matthew J Lee, Thomas D Pinkney

**Affiliations:** Department of Applied Health Sciences, College of Medicine and Health, University of Birmingham, Birmingham, UK; Department of Applied Health Sciences, College of Medicine and Health, University of Birmingham, Birmingham, UK

Surgical site infection (SSI) and delayed wound healing are problematic to all surgeons. They result in pain, delayed recovery, and increased healthcare costs both in the hospital and the community. It is therefore not surprising that surgeons and the healthcare industry devote a significant amount of time and effort to reducing the burden from this largely preventable complication.

Since the first reports almost 30 years ago^1^, negative pressure wound therapy (NPWT) appeared a promising tool in the armamentarium offering a mechanistically plausible means of improving wound health and healing. The intervention purports to reduce the risk of SSI by withdrawing exudate out of a wound through suction into a dressing pad, as well as perhaps enhancing local blood flow. The negative pressure is also thought to stimulate fibroblastic activity, together leading to granulation and accelerated tissue healing, a particular benefit for wounds healing by secondary intention. A Cochrane review was published in 2015 but could only identify limited robust evidence of clinical or cost-effectiveness, the authors advising caution when interpreting the findings^2^.

Over the past decade and a half, there has been an explosion of interest in using negative pressure wound therapy on *closed* surgical wounds healing by primary intention, often termed incisional negative pressure wound therapy (iNPWT). Multiple small RCTs in this context have been published across a variety of operative procedures, and their findings reviewed and meta-analysed several times, with conflicting conclusions. The most recent (fourth update) Cochrane review was published in 2022 demonstrating a maturing of the evidence^3^. In that review, 44 studies (11 403 people) reporting SSI outcomes were pooled. It concluded that there was ‘moderate-certainty evidence that NPWT probably results in fewer SSIs compared with standard dressings’. However, the risks of bias across the included studies caused the evidence to be downgraded. It may also be relevant to note that almost half of trials included in the review were funded by industry. The authors noted that there was a large number of ongoing studies, the results of which could change the findings of the review.

Recent weeks have seen the publication of two such multicentre RCTs in NPWT and iNPWT, both funded by the UK National Institute for Health and Care Research (NIHR). The SUNRRISE trial explored the clinical effectiveness of iNPWT to reduce SSI after emergency laparotomy^4^. It recruited 840 participants from 34 units across the UK and Australia and showed that the intervention did not influence SSI rates.

In contrast, Surgical Wounds Healing by Secondary Intention (SWHSI-2) evaluated NPWT use in wounds left to heal by secondary intention, mostly across the foot and leg. This trial showed no benefit from the use of NPWT in terms of time to wound healing across 686 participants from 29 sites^5^. The associated health economic analysis of SWHSI-2, published in *BJS*, did not identify any evidence of cost-effectiveness compared to standard dressings^6^.

What do these findings mean for us in practice? No trial is perfect, and people will always be able to pick potential holes in published evidence—for example, to ask if the trial assessed the correct device, at the correct suction pressure, for the duration of intervention to be able to identify a potential benefit. There are also the inevitable inbuilt vagaries around the design, conduct, and analysis of any multicentre trial. A pragmatist might argue that NPWT does not hurt and so its use might perhaps be justified, but these devices are not cheap and, in a resource-constrained healthcare setting, this is money that could be used more effectively elsewhere if these devices do not deliver on their desired benefits.

So are we at the end of a long, painful road where NPWT has ultimately been shown to be no more effective and more costly compared with standard approaches to managing wounds in surgery? Perhaps not. Although it is too soon to know if these newly published trials will result in widespread de-adoption of NPWT and iNPWT, it is interesting to note that several new manufacturers have released their own NPWT or iNPWT devices onto the market over the past couple of years. The devices benefit from ‘making sense’ to surgeons and patients alike in terms of *how* and *why* they might work to deliver improved wound outcomes, and as such it seems likely that we will require very robust evidence of non-effectiveness before surgeons, nurses, and patients alike are willing to give them up. The WHO SSI prevention guidelines from 2018 made a conditional recommendation for the use of iNPWT ‘in patients at high-risk of SSI’, a policy which is also supported by NICE Medical Technology Guidance from 2019^7,8^. These will therefore not be the last trials published in this area.

Surgical wounds are not all alike, and evidence on what works (or does not work) in one wound type or specialty area is not necessarily translatable to others. The world needs robust and context-specific assessment of this technology to allow informed decisions to be made. The recent NIHR-funded major ROSSINI-Platform trial will explore the reduction of SSI across the body by running multiple parallel trials in six initial clinical specialties or ‘pillars’ under one overarching protocol and delivery network^9^. Several of these pillar teams have identified iNPWT as one of their key interventions demonstrating an evidence gap, community equipoise, and the need for high-quality testing within their specific clinical area.

Watch this space.
